# Pretreatment Lactate Dehydrogenase-to-Albumin Ratio and Clinical Outcomes in Extensive-Stage Small Cell Lung Cancer: A Multicenter Real-World Study

**DOI:** 10.3390/jcm15093353

**Published:** 2026-04-28

**Authors:** Ahmet Unlu, Asim Armagan Aydin, Esra Sazimet Kars, Ozden Ozturk, Mehmet Acun, Mehmet Nuri Baser, Mahmut Kara, Sati Sena Coraoglu, Nurbanu Inci, Muhammet Ali Kaplan, Bilgin Demir, Senar Ebinc, Okan Avci, Hacer Boztepe Yesilcay, Banu Ozturk, Mustafa Yildiz

**Affiliations:** 1 Department of Medical Oncology, Antalya Training and Research Hospital, University of Health Sciences, 07100 Antalya, Turkey; drarmaganaydin@gmail.com (A.A.A.); mehmetacun07@hotmail.com (M.A.); drbanutr@yahoo.com (B.O.); drmyildiz@yahoo.com (M.Y.); 2 Department of Medical Oncology, Dicle University Faculty of Medicine, 21100 Diyarbakir, Turkeydrkaplan@gmail.com (M.A.K.); 3 General Directorate of Public Health, Genetic Diseases Screening Laboratory, Ministry of Health, 06100 Ankara, Turkey; 4 Department of Medical Oncology, Aydin Adnan Menderes University Faculty of Medicine, 09100 Aydin, Turkey; 5 Department of Medical Oncology, Dursun Odabas Medical Center, Van Yuzuncu Yil University, 65100 Van, Turkey; 6 Department of Medical Oncology, Faculty of Medicine, Tekirdag Namik Kemal University, 59100 Tekirdag, Turkey; 7 Department of Medical Oncology, Ataturk Training and Research Hospital, Izmir Katip Celebi University, 35100 Izmir, Turkey; nurbanu_inci@hotmail.com; 8 Department of Thoracic Surgery, Antalya Training and Research Hospital, University of Health Sciences, 07100 Antalya, Turkey

**Keywords:** LDH-to-albumin ratio, lactate dehydrogenase, small-cell lung cancer, extensive stage, treatment failure, prognosis, LAR, survival, immune inflammation, albumin

## Abstract

**Background:** Reliable biomarkers that capture tumor–host interactions and predict treatment resistance in extensive-stage small cell lung cancer (SCLC) remain limited. We evaluated the prognostic and predictive value of the pretreatment lactate dehydrogenase-to-albumin ratio (LAR), an integrative biomarker reflecting metabolic activity, systemic inflammation, and host nutritional status. **Methods:** This multicenter, retrospective cohort study included patients with extensive-stage SCLC treated at five tertiary centers between 2016 and 2024. Pretreatment LAR was calculated using baseline serum lactate dehydrogenase and albumin levels and dichotomized using a Youden index-derived cut-off at the 12-month overall survival (OS) horizon. Time-dependent receiver operating characteristic (ROC) analyses using inverse probability weighting were performed to assess discriminative performance. Survival outcomes were evaluated using Kaplan–Meier estimates and Cox proportional hazards models. Associations with platinum resistance and lack of objective treatment benefit (defined as progressive disease as best response) were examined using logistic regression models. **Results:** A total of 223 patients were included. Elevated LAR was associated with inferior OS (median, 15.8 vs. 25.2 months; log-rank *p* < 0.001) and progression-free survival (7.9 vs. 11.5 months; *p* < 0.001). In multivariable analysis, LAR remained independently associated with OS (HR, 1.43; 95% CI, 1.04–1.95; *p* = 0.028). LAR demonstrated modest but consistently superior discriminative performance compared with other inflammatory indices for both 12-month OS (area under the curve [AUC], 0.692) and 6-month progression-free survival (PFS) (AUC, 0.646), with statistically significant differences in DeLong comparisons. Higher LAR was independently associated with increased odds of platinum resistance (adjusted odds ratio [aOR], 2.31; 95% CI, 1.41–3.81; *p* = 0.001) and lack of objective treatment benefit (adjusted OR, 2.04; 95% CI, 1.33–3.14; *p* = 0.001). **Conclusions:** Pretreatment LAR is a clinically accessible and biologically integrative biomarker associated with survival and treatment resistance in extensive-stage SCLC. By capturing tumor–host interactions, LAR may support risk stratification and identify patients at increased risk of early treatment failure. Prospective validation is warranted to define its role in biomarker-driven clinical decision-making.

## 1. Introduction

Small cell lung cancer (SCLC) is a highly aggressive neuroendocrine malignancy that accounts for approximately 13–15% of all lung cancers and remains a major contributor to cancer-related mortality worldwide [1,2,3]. Despite representing a minority of lung cancer cases, SCLC is characterized by a rapidly progressive clinical course, early dissemination, and limited durability of treatment response [4]. At the molecular level, it is defined by high proliferative activity, genomic instability, and frequent inactivation of key tumor suppressor pathways, contributing to its aggressive phenotype [5,6]. Clinically, although SCLC demonstrates high initial sensitivity to cytotoxic chemotherapy, most patients experience early relapse, particularly in the extensive-stage setting, which constitutes the majority of cases at diagnosis [7]. The addition of immune checkpoint inhibitors to platinum-based chemotherapy has led to modest improvements in survival [8]; however, outcomes remain heterogeneous, and early treatment resistance is common [9]. This variability underscores the critical unmet need for clinically accessible biomarkers capable of identifying patients at increased risk of treatment failure and poor survival.

In addition to tumor-intrinsic characteristics, systemic inflammation, metabolic stress, and host nutritional status are increasingly recognized as key determinants of cancer progression and therapeutic response [10,11]. Blood-based biomarkers derived from routine laboratory parameters, such as the neutrophil-to-lymphocyte ratio (NLR) and related inflammatory indices, have shown prognostic value across multiple malignancies, including SCLC [12,13,14]. However, these markers largely reflect isolated components of the host response and may not adequately capture the complex interplay between tumor biology and systemic host vulnerability. Therefore, there remains a need for integrative biomarkers that better represent the multidimensional nature of tumor–host interactions.

The lactate dehydrogenase-to-albumin ratio (LAR) is a biologically grounded and clinically accessible biomarker that integrates key dimensions of tumor metabolism and host physiology. Lactate dehydrogenase (LDH) reflects enhanced glycolytic flux and hypoxia-driven metabolic reprogramming, hallmarks of aggressive tumor behavior that promote rapid proliferation, immune evasion, and resistance to therapy through lactate-mediated microenvironmental changes [15,16,17]. In contrast, albumin serves as a surrogate of systemic inflammation and nutritional status, reflecting a host milieu characterized by cytokine-driven catabolism, impaired immune competence, and reduced physiological reserve [18]. By combining these parameters, LAR captures a dynamic tumor–host interface shaped by metabolic stress and systemic inflammatory signaling [19,20]. This integrative framework may provide a more biologically meaningful representation of disease aggressiveness and treatment resistance than conventional inflammation-based indices that assess isolated components of the host response.

In this context, we conducted a multicenter, real-world cohort study to evaluate the prognostic and predictive value of pre-treatment LAR in patients with extensive-stage SCLC. Specifically, we assessed its association with survival outcomes, discriminative performance relative to established inflammatory indices using time-dependent analytical approaches, and relationship with clinically relevant treatment-related endpoints, including platinum resistance and treatment response patterns.

## 2. Materials and Methods

### 2.1. Study Design and Patient Selection

This multicenter, real-world, retrospective cohort study was conducted within the Oncology-Network (ONCONET) Collaborative Group across five tertiary oncology centers in Turkey (Antalya, Diyarbakir, Aydin, Van, and Tekirdag). We screened consecutive patients with histologically or cytologically confirmed extensive-stage SCLC treated between August 2016 and December 2024. This study aimed to evaluate the clinical relevance of pretreatment blood-based biomarkers, with a particular focus on the LAR, in relation to survival and treatment outcomes.

Eligible patients had extensive-stage disease at diagnosis, received first-line systemic therapy, and had available baseline LDH and albumin measurements obtained before treatment initiation. To reduce confounding, patients were excluded if they had recent infection-related treatments (antibiotics or corticosteroids within 4 weeks), blood transfusion within 6 weeks, albumin or plasma administration within 4 weeks, prior palliative radiotherapy, synchronous malignancy, use of alternative therapies before diagnosis, or missing baseline data.

The primary endpoint was overall survival (OS), defined as the time from treatment initiation to death from any cause. Secondary endpoints included progression-free survival (PFS), treatment response, platinum resistance, and lack of objective treatment benefit.

**Figure 1:**
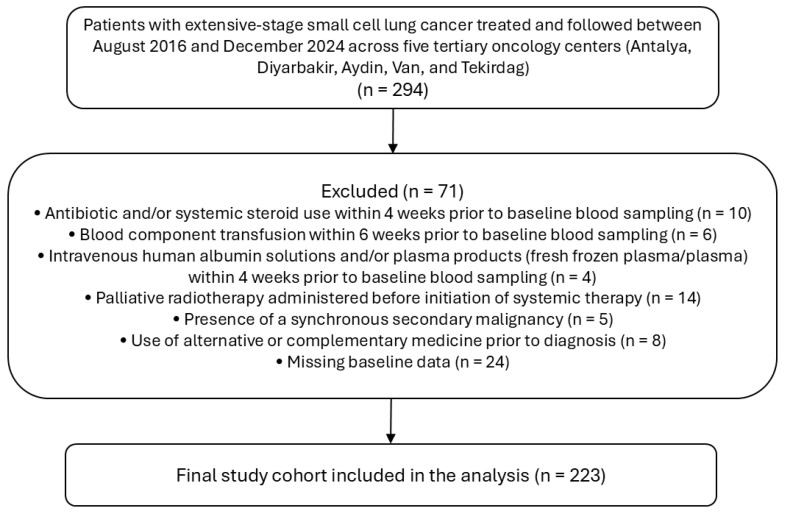
Flow diagram of patient selection and eligibility.

Of the 294 screened patients, 223 met the eligibility criteria and were included in the final analysis (Figure 1).

This study was conducted in accordance with the Declaration of Helsinki and approved by the Institutional Review Board of the University of Health Sciences Antalya Training and Research Hospital and the Antalya Provincial Health Directorate (approval no. 2-14; 22 January 2026). The requirement for informed consent was waived owing to the retrospective design and use of anonymized data.

### 2.2. Data Collection and Variable Definitions

Clinical, demographic, treatment, and laboratory data were extracted from electronic medical records using a standardized framework harmonized across participating centers. Baseline variables included age, sex, smoking status, Eastern Cooperative Oncology Group (ECOG) performance status, comorbidities, and metastatic sites at diagnosis, with particular emphasis on brain and liver involvement. Tumor laterality and lobar location were also recorded. Treatment-related variables included first-line systemic therapy characteristics and platinum backbone.

Pretreatment laboratory parameters were obtained from routine blood tests performed within 7 days prior to the initiation of first-line therapy and included complete blood count and biochemical measurements, with particular emphasis on LDH and albumin levels. Additional inflammation- and nutrition-related indices were derived for comparative analyses.

Tumor response was assessed according to the Response Evaluation Criteria in Solid Tumors (RECIST), version 1.1 (RECIST v1.1). Best overall response was categorized as complete response (CR), partial response (PR), stable disease (SD), or progressive disease (PD). Objective response (ORR) was defined as CR or PR, whereas disease control (DCR) was defined as CR, PR, or SD. Lack of objective treatment benefit was defined as PD as the best response to first-line therapy.

OS was defined as the time from the initiation of first-line systemic therapy to death from any cause. PFS was defined as the time from treatment initiation to radiologic or clinical disease progression or death, whichever occurred first. Patients without an event were censored at the date of the last follow-up. Censoring was primarily administrative at the predefined study cutoff (December 2024). A minority of patients were censored due to loss to follow-up, including transfer of care or non-attendance.

Platinum resistance was defined as disease progression occurring during or within 6 months after completion of platinum-based chemotherapy. In accordance with clinical practice in extensive-stage SCLC, patients who died due to rapid disease progression within this 6-month window, prior to formal radiologic reassessment, were classified as platinum-resistant, as such cases are considered to reflect primary refractory disease biology. Deaths occurring within this interval were reviewed at each center, and all early deaths were attributable to disease progression rather than non–cancer-related causes. Therefore, all patients could be unambiguously classified as either platinum-sensitive or platinum-resistant based on the predefined criteria.

The index date for all time-to-event analyses was defined as the initiation of first-line systemic therapy.

### 2.3. Biomarker Definitions

The primary biomarker was the pretreatment LAR, calculated as (LDH, U/L) divided by albumin (g/dL). Comparator indices included NLR [12], monocyte-to-lymphocyte ratio (MLR) [21], platelet-to-lymphocyte ratio (PLR) [22], systemic immune-inflammation index (SII) [23], systemic inflammatory response index (SIRI) [24], pan-immune-inflammation value (PIV) [25], C-reactive protein-to-albumin ratio (CAR) [26], and monocyte-to-albumin ratio (MAR) [27], which were calculated using standard formulas.

LAR was analyzed as a continuous variable and dichotomized using an optimal cutoff derived from receiver operating characteristic (ROC) analysis at the 12-month OS horizon using the Youden index. This cutoff was applied consistently across all analyses.

For exploratory analyses of treatment-related endpoints, separate ROC analyses were conducted for each endpoint, and corresponding endpoint-specific Youden-derived cutoffs were reported to assess context-specific discriminatory performance.

The definitions and formulas of the biomarker indices are provided in Supplementary Table S1.

### 2.4. Treatment Protocol and Clinical Follow-Up

Patients underwent first-line therapy in accordance with institutional practice and contemporary guidelines. Platinum-based chemotherapy (cisplatin or carboplatin) was used in most cases. Given the multicenter retrospective design, variations in treatment regimens and supportive care were expected and reflected real-world practice. Patients were followed up according to institutional protocols with periodic clinical and radiologic assessments. Imaging was interpreted locally by experienced radiologists.

### 2.5. Statistical Analysis

All statistical analyses were performed using R (version 4.4.3; R Foundation for Statistical Computing, Vienna, Austria) and SPSS (version 27.0; IBM Corp., Armonk, NY, USA). Two-sided *p* values < 0.05 were considered statistically significant. Categorical variables were compared using the χ^2^ or Fisher’s exact test, and continuous variables were summarized as medians with interquartile ranges.

The discriminative performance of the LAR and comparator indices was assessed using time-dependent ROC analyses with inverse probability of censoring weighting (IPCW), focusing on 12-month OS and 6-month PFS. Cut-off values were derived using the Youden index at the 12-month OS horizon and applied across endpoints. AUC comparisons were performed using the DeLong test.

For treatment-related endpoints, additional exploratory ROC analyses were performed, and endpoint-specific cutoffs were derived using the Youden index to evaluate the discriminatory performance of LAR in a context-dependent manner.

Survival was analyzed using Kaplan–Meier estimates and log-rank tests. Cox proportional hazards models were used for univariable and multivariate analyses. Multivariate models included prespecified covariates (age, ECOG performance status, brain metastasis, and treatment response). Model C included LAR, whereas Model D additionally incorporated MAR to explore the overlap between the inflammation–nutrition indices.

Associations with treatment-related endpoints were evaluated using logistic regression, adjusted for age, ECOG performance status, and brain metastasis.

Biomarkers were analyzed as continuous and dichotomized variables to balance statistical rigor with clinical interpretability.

## 3. Results

### 3.1. Baseline Characteristics of the Study Cohort

In the final analysis, 223 patients with extensive-stage SCLC were included. The age distribution of the cohort was comparable across strata, with 113 patients (50.7%) younger than 65 years and 110 patients (49.3%) aged ≥65 years. Most patients were men (*n* = 193, 86.5%), consistent with the known epidemiology of extensive-stage disease. At diagnosis, most patients had a preserved functional status, with 145 patients (65.0%) presenting with an ECOG performance status of 0–1, while 77 patients (35.0%) had an ECOG performance status of ≥2.

Regarding smoking history, 35 patients (15.7%) were never-smokers, 121 (54.3%) were former smokers, and 67 (30.0%) were current smokers. Comorbid conditions were present in 136 patients (61.0%). At baseline staging, brain metastases were observed in 82 patients (36.8%), and liver metastases were identified in 91 patients (40.8%), indicating a substantial metastatic burden at diagnosis.

Primary tumors were slightly more frequently left-sided (55.2%), and upper-lobe involvement represented the most common primary tumor location. Nearly all patients received platinum-based chemotherapy in the first-line setting, with cisplatin administered in 65.5% and carboplatin in 32.3% of cases. Disease control (DCR)—defined as CR, PR, or SD—was achieved in 160 patients (71.7%), whereas 63 patients (28.3%) experienced PD as the best response. Platinum resistance developed in 95 patients (42.6%) during the disease course. Baseline demographic, clinical, treatment-related, and inflammatory characteristics of the cohort are summarized in Table 1.

### 3.2. Baseline Characteristics According to Pretreatment LDH-to-Albumin Ratio

A prespecified Youden index-derived cut-off value of 65.37 was used to stratify patients into low- and high-LAR groups (*n* = 97 and *n* = 126, respectively). Demographic variables, including age and sex, were comparable between the two groups. However, smoking status differed significantly, with a lower proportion of never-smokers in the high-LAR group (*p* = 0.037). Functional status also tended to be less favorable among patients with high LAR, with a numerically higher frequency of ECOG performance status ≥2.

Distinct differences were observed in metastatic patterns at diagnosis. The presence of liver metastasis was substantially more frequent in the high-LAR group than in the low-LAR group (58.3% vs. 17.5%; *p* < 0.001). In contrast, brain metastases at diagnosis were less commonly observed among patients with high LAR (28.3% vs. 47.4%; *p* = 0.010). Primary tumor laterality, lobar distribution, and the choice of platinum backbone in the first-line setting were otherwise similar between the LAR strata (Table 1).

Treatment response profiles differed significantly according to LAR status. Patients in the high-LAR group were more likely to experience PD as the best response to first-line therapy and demonstrated a lower rate of disease control compared to those in the low-LAR group (64.6% vs. 80.4%; *p* = 0.028). Platinum resistance occurred more frequently in the high-LAR group (49.6% vs. 33.0%; *p* = 0.027). Among patients with available follow-up data, the incidence of brain metastasis at progression did not differ significantly between the LAR groups (Table 1).

Pretreatment LAR status was also closely associated with a systemic inflammatory burden. Elevated levels of NLR, MLR, PLR, SII, SIRI, PIV, CAR, and MAR—each dichotomized using ROC–Youden index-derived cut-off values—were markedly more prevalent in the high-LAR group (all *p* < 0.001), indicating a distinct inflammatory phenotype associated with elevated LAR.

### 3.3. Time-Dependent ROC Analyses of Inflammatory Indices

Time-dependent ROC analyses using inverse probability of censoring weighting were performed to evaluate the discriminative performance of pretreatment inflammatory indices for clinically relevant time horizons, focusing on 12-month OS and 6-month PFS. For each index, optimal cutoff values were derived from the 12-month OS horizon using the Youden index and were subsequently applied consistently across endpoints.

**Figure 2:**
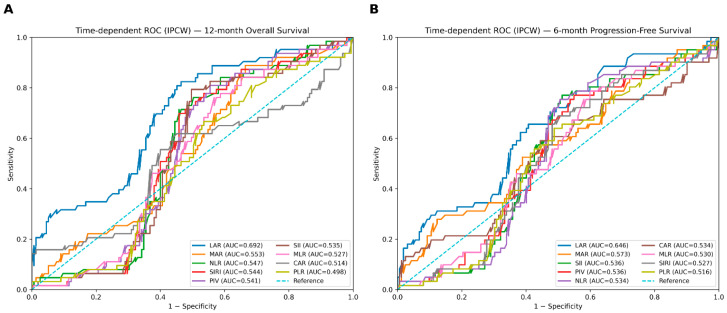
Time-dependent ROC analysis of inflammatory indices in extensive-stage small cell lung cancer. (**A**) Time-dependent ROC curves for 12-month overall survival (OS). (**B**) Time-dependent ROC curves for 6-month progression-free survival (PFS).

At the 12-month OS horizon, LAR demonstrated the numerically highest discriminative performance among all evaluated indices, with an AUC(t) of 0.692 (Figure 2A). The corresponding Youden-derived cutoff value was 65.37, yielding a sensitivity of 0.825 and a specificity of 0.535 (Table 2). The remaining inflammatory indices showed more modest discrimination, with AUC(t) values clustering around 0.50–0.55.

In the time-dependent ROC analysis for 6-month PFS, LAR achieved the highest AUC(t) value (0.646), supporting its superior discrimination for early disease progression relative to the comparator indices (Figure 2B; Table 2). Comparative analyses using the DeLong test, with LAR as the reference, confirmed that the AUC values for LAR at the 12-month OS horizon were significantly higher than those of each comparator index (Table 2).

### 3.4. Kaplan–Meier Survival Analyses According to LAR

**Figure 3:**
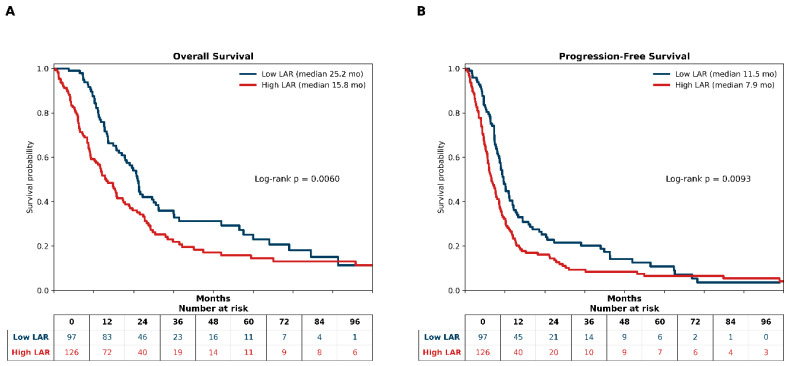
Kaplan–Meier survival curves according to LDH-to-albumin ratio (LAR) in extensive-stage small cell lung cancer. (**A**) Overall survival (OS); (**B**) progression-free survival (PFS). Patients were stratified using the 12-month OS Youden index–derived LAR cutoff. Survival distributions were compared using the log-rank test.

At a median follow-up of 15.2 months (interquartile range, 9.1–25.6), the median OS for the overall cohort was 20.4 months (95% CI, 16.3–24.8), and the median PFS was 9.9 months (95% CI, 8.8–11.1). Kaplan–Meier analyses stratified by pretreatment LAR demonstrated a clear separation between the low- and high-LAR groups for both survival endpoints (Figure 3).

Patients in the low-LAR group experienced significantly prolonged OS compared with those in the high-LAR group, with median OS values of 25.2 months (95% CI, 21.4–30.7) and 15.8 months (95% CI, 12.6–20.9), respectively (log-rank *p* < 0.001). Similarly, the median PFS was significantly longer in the low-LAR group than in the high-LAR group (11.5 months [95% CI, 10.4–14.2] vs. 7.9 months [95% CI, 6.9–10.2]; log-rank *p* < 0.001). Number-at-risk tables accompanying each Kaplan–Meier plot illustrate the temporal distribution of patients across the LAR strata throughout the follow-up.

### 3.5. Univariate Cox Proportional Hazards Analyses

Univariate Cox proportional hazards analyses were performed to evaluate the associations between baseline clinical characteristics, metastatic features, treatment-related variables, systemic inflammatory indices, and survival outcomes (Table 3).

Among inflammatory and metabolic biomarkers, pretreatment LAR demonstrated a robust association with both OS and PFS. High LAR was associated with an increased risk of death (HR, 1.563; 95% CI, 1.148–2.128; *p* = 0.005) and disease progression (HR, 1.467; 95% CI, 1.108–1.943; *p* = 0.008). A high MAR was also associated with inferior outcomes for OS (HR, 1.484; 95% CI, 1.046–2.105; *p* = 0.027) and PFS (HR, 1.387; 95% CI, 1.012–1.900; *p* = 0.042).

Treatment-related variables were strong prognostic determinants. Achieving disease control (SD/PR/CR) versus PD was associated with substantially reduced hazards for both OS (HR, 0.319; 95% CI, 0.230–0.443; *p* < 0.001) and PFS (HR, 0.267; 95% CI, 0.195–0.366; *p* < 0.001). Conversely, platinum resistance was associated with markedly increased risks of death (HR, 3.991; 95% CI, 2.902–5.490; *p* < 0.001) and progression (HR, 5.861; 95% CI, 4.248–8.088; *p* < 0.001).

### 3.6. Multivariable Cox Regression Analyses

Multivariable Cox proportional hazards models were constructed to assess the independent prognostic value of LAR after adjusting for clinically relevant covariates (Table 4). Model C included age, ECOG performance status, brain metastasis at diagnosis, treatment response, and LAR. Model D additionally incorporated MAR to explore potential overlap with other nutrition-inflammation indices.

In Model C, high LAR remained independently associated with inferior OS after adjustment for clinical characteristics and treatment response (HR, 1.425; 95% CI, 1.040–1.954; *p* = 0.028). When MAR was added to the model (Model D), the effect estimate for LAR was attenuated and shifted toward borderline statistical significance, whereas the direction of the effect was preserved.

For PFS, treatment response emerged as the dominant independent determinant across models. In contrast, LAR did not retain an independent association with PFS in multivariate analyses, indicating that its prognostic impact was primarily related to long-term survival rather than early disease progression.

### 3.7. Association of Pretreatment LAR with Treatment Response and Platinum Resistance

**Figure 4:**
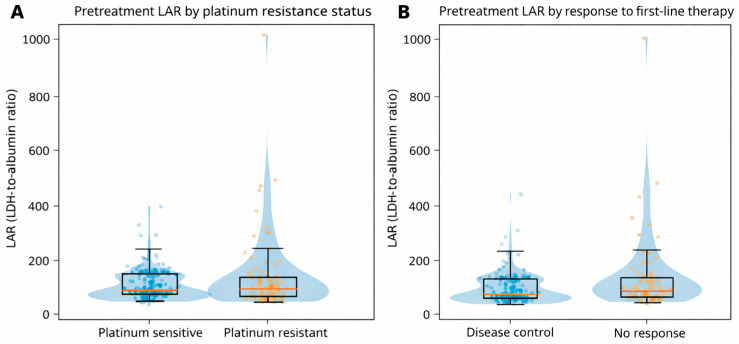
Distribution of pretreatment LDH-to-albumin ratio according to adverse treatment-related features in extensive-stage small cell lung cancer. Violin plots illustrate the distribution of pretreatment LDH-to-albumin ratio (LAR) values, with embedded box plots indicating the median and interquartile range; individual dots represent individual patients. The blue and orange colors represent the respective comparison groups in each panel. (**A**) shows the LAR distribution according to platinum resistance status. (**B**) shows the LAR distribution according to objective treatment benefit from first-line therapy (disease control vs. no response).

In exploratory analyses, higher pretreatment LAR values were associated with adverse treatment-related features (Figure 4; Table 5). LAR demonstrated modest discriminatory ability for platinum resistance (AUC, 0.615) and for lack of objective treatment benefit from first-line therapy (AUC, 0.648).

In multivariable logistic regression models adjusted for age, ECOG performance status, and presence of brain metastasis at diagnosis, increased LAR was independently associated with a higher odds of platinum resistance (adjusted OR, 2.31; 95% CI, 1.41–3.81; *p* = 0.001) and lack of objective treatment benefit (adjusted OR, 2.04; 95% CI, 1.33–3.14; *p* = 0.001).

## 4. Discussion

In this multicenter real-world cohort of patients with extensive-stage SCLC, pretreatment LAR emerged as a clinically informative and biologically grounded biomarker associated with both survival outcomes and treatment-related endpoints. Elevated LAR identified a subgroup of patients with inferior OS, a lower likelihood of achieving disease control, and a higher risk of platinum resistance. Notably, these associations were consistent across multiple analytical approaches, including time-dependent discrimination analyses, survival modeling, and regression-based evaluations of treatment outcomes. Collectively, these findings suggest that LAR captures a composite tumor–host biological state linking metabolic activity, systemic inflammation, and host reserve, rather than functioning solely as a conventional prognostic marker.

SCLC is characterized by rapid proliferation, metabolic reprogramming, and early dissemination [6]; however, biomarkers that capture the interplay between tumor aggressiveness and host vulnerability remain limited [7]. In this context, LAR represents an integrative marker linking tumor metabolic activity with systemic host responses. Lactate dehydrogenase, a key enzyme in anaerobic glycolysis, reflects enhanced glycolytic flux consistent with the Warburg phenotype and has been associated with tumor burden, hypoxia adaptation, and adverse outcomes across solid tumors [15,28]. In contrast, serum albumin, a negative acute-phase reactant, captures systemic inflammation, cancer-related catabolism, and impaired host reserve—processes closely linked to immune dysregulation and treatment tolerance [29]. By combining these complementary dimensions, LAR reflects a tumor–host axis shaped by metabolic stress and systemic inflammation, which may underlie its ability to stratify clinically distinct phenotypes beyond conventional inflammatory indices [19].

A key finding of the present study is the consistent, albeit moderate, discriminative performance of LAR compared with widely used inflammatory indices. In time-dependent ROC analyses accounting for censoring through inverse probability weighting, LAR consistently demonstrated the highest discrimination for both 12-month OS and 6-month PFS whereas comparator indices showed limited performance, with AUC values clustering near the non-informative range. Importantly, discrimination was evaluated at clinically meaningful time horizons, and cutoff values were derived using a prespecified Youden index approach at the 12-month overall survival landmark and applied consistently across endpoints. Comparative analyses using the DeLong test further confirmed that LAR provided significantly greater discriminative ability than each of the evaluated comparator indices. These findings support the robustness of LAR as a prognostic tool and suggest that integrative biomarkers capturing tumor–host interactions may offer greater clinical utility than conventional inflammation-based measures.

Survival analyses further reinforced the clinical relevance of LAR. Kaplan–Meier estimates demonstrated a clear and sustained separation between LAR-defined strata for both OS and PFS, supporting its ability to distinguish clinically meaningful risk groups. In univariable analyses, elevated LAR was consistently associated with an increased hazard of death and disease progression, alongside established treatment-related determinants, such as response status and platinum resistance. In multivariable models adjusting for key clinical factors, including performance status, metastatic burden, and treatment response (Model C), LAR remained independently associated with OS, underscoring its prognostic robustness beyond conventional clinical parameters. Notably, the inclusion of MAR in an extended model (Model D) attenuated the effect estimate of LAR, with preservation of the direction of the effect. Rather than indicating redundancy, this pattern suggests a partial biological overlap among inflammation–nutrition indices, reflecting shared yet non-identical dimensions of tumor–host interaction. Consistent with this interpretation, LAR did not retain independent significance for progression-free survival in adjusted analyses, suggesting that its primary contribution lies in capturing longer-term survival dynamics rather than early disease progression.

In addition to its prognostic relevance, elevated LAR was significantly associated with treatment-related outcomes, including platinum resistance and lack of objective treatment benefit, defined as progressive disease as the best response to first-line therapy. Patients with elevated LAR exhibited higher rates of disease progression and an increased likelihood of platinum-refractory disease. These relationships remained significant after adjustment for key clinical variables in multivariable models, supporting their independence from established prognostic factors. Although the discriminatory performance of LAR for these endpoints was modest, the consistency of these findings across multiple analytical approaches reinforces their robustness. Collectively, this pattern suggests that LAR may reflect an underlying biological state associated with primary treatment refractoriness, potentially driven by metabolic stress and systemic inflammation. In a disease where platinum-based chemotherapy remains the therapeutic backbone, the ability to identify patients at increased risk of early treatment failure using a simple pretreatment biomarker may have meaningful clinical implications. However, this interpretation warrants caution. In patients with aggressive disease, early death due to rapid progression may act as a competing event that precludes formal radiologic confirmation of progression, thereby introducing potential classification bias. Within this framework, the relationship between LAR and platinum resistance is more appropriately interpreted as reflecting an underlying biological susceptibility to early treatment failure rather than a strictly time-dependent predictive effect.

The observed association between elevated LAR and metastatic patterns further supports its role as a marker of aggressive disease biology. Patients with high LAR more frequently presented with liver metastases, a clinical feature consistently linked to high tumor burden, enhanced metabolic activity, and adverse prognosis in SCLC. This enrichment aligns with the biological dimensions captured by LAR, particularly tumor-driven metabolic stress and systemic inflammatory activation. In contrast, brain metastases at diagnosis were less frequent among patients with elevated LAR, suggesting that distinct metastatic trajectories may underlie biologically heterogeneous subsets of extensive-stage disease. Although these findings should be interpreted with caution, they raise the possibility that LAR may reflect differences in metastatic tropism shaped by tumor–host interactions. This observation further supports the concept that LAR captures a broader biological phenotype beyond conventional prognostic markers, encompassing not only disease burden but also patterns of dissemination.

From a clinical perspective, LAR offers several practical advantages as a biomarker in extensive-stage SCLC. It is derived from routinely available laboratory parameters, requires no additional cost or specialized infrastructure, and can be readily implemented across diverse clinical settings. Importantly, its association with both survival outcomes and treatment resistance suggests potential utility not only for prognostic stratification but also for identifying patients at a higher risk of early treatment failure. In this context, LAR may support a more refined risk assessment at baseline and could be considered in the design of risk-adapted therapeutic strategies or clinical trial stratification frameworks. Furthermore, as an integrative tumor–host biomarker, LAR may complement existing clinical and radiologic parameters by providing additional biological context that is not captured by conventional staging systems alone.

This study has several limitations that should be considered. First, the retrospective design introduces the possibility of residual confounding and selection bias, despite the use of predefined eligibility criteria and multivariate adjustment strategies. Second, the inherent heterogeneity of real-world treatment patterns, including differences in platinum backbone and supportive care, may have influenced clinical outcomes and treatment-related endpoints. Although the multicenter nature of the cohort enhances generalizability, external validation in independent and prospectively collected datasets is required to confirm the reproducibility and generalizability of these findings. Early death may represent a competing event that influences the classification of platinum resistance, particularly in patients with rapidly progressive disease. As formal competing risks analyses were not feasible within the retrospective design, this potential source of bias should be considered when interpreting the observed associations. Accordingly, the relationship between LAR and platinum resistance should be regarded as hypothesis-generating. In addition, the present analysis was based on baseline biomarker measurements, and longitudinal changes in LAR during treatment were not evaluated; such dynamic assessments may provide further insight into treatment response, resistance evolution, and tumor–host adaptation over time. From a translational perspective, the biological mechanisms linking elevated LAR to treatment refractoriness remain incompletely understood and warrant further investigation, particularly in relation to metabolic reprogramming, systemic inflammation, and immune dysregulation. Future studies integrating LAR with molecular, genomic, and microenvironmental data may help refine its biological interpretation and clinical applicability. Prospective validation and incorporation of LAR into biomarker-driven clinical trial designs will be essential to determine whether its use can meaningfully inform therapeutic decision-making in ES-SCLC.

## 5. Conclusions

In this multicenter real-world cohort, pretreatment LAR delineates a clinically and biologically distinct phenotype of extensive-stage SCLC characterized by inferior survival and increased susceptibility to treatment resistance. By integrating markers of tumor metabolic activity and host systemic vulnerability, LAR captures a composite tumor–host state that is not fully reflected by conventional inflammatory indices. Although its discriminative performance is moderate, the consistency of its associations across survival and treatment-related endpoints suggests that LAR encodes a reproducible signal of disease aggressiveness rather than isolated prognostic information. In particular, its link with platinum resistance and lack of objective treatment benefit points to a biologically relevant substrate of early therapeutic failure. Taken together, these findings position LAR as a promising, hypothesis-generating biomarker within a tumor–host interaction framework. Prospective validation will be required to determine whether this integrative signal can be leveraged to inform risk-adapted therapeutic strategies in extensive-stage SCLC.

## Figures and Tables

**Table 1:** Baseline Demographic, Clinical, Treatment-Related, and Inflammatory Characteristics of Patients With Extensive-Stage Small-Cell Lung Cancer According to Pretreatment LDH-to-Albumin Ratio (LAR) (*n* = 223).

Variable	Category	Overall, *n* (%)	LDH-to-Albumin Ratio (LAR)		
			Low LAR, *n* (%)	High LAR, *n* (%)	*p*
Age	<65	113 (50.7)	50 (51.5)	63 (49.6)	0.930
	≥65	110 (49.3)	47 (48.5)	63 (49.6)	
Sex	Male	193 (86.5)	87 (89.7)	106 (83.5)	0.329
	Female	30 (13.5)	10 (10.3)	20 (15.7)	
ECOG PS	0–1	145 (65.0)	69 (71.9)	76 (59.8)	0.137
	≥2	77 (35.0)	27 (28.1)	50 (39.4)	
Smoking status	Never	35 (15.7)	22 (22.7)	13 (10.2)	0.037
	Former	121 (54.3)	50 (51.5)	71 (55.9)	
	Current	67 (30.0)	25 (25.8)	42 (33.1)	
Comorbidity	No	87 (39.0)	33 (34.0)	54 (42.5)	0.277
	Yes	136 (61.0)	64 (66.0)	72 (56.7)	
Brain metastasis at diagnosis	No	141 (63.2)	51 (52.6)	90 (70.9)	0.010
	Yes	82 (36.8)	46 (47.4)	36 (28.3)	
Liver metastasis at diagnosis	No	132 (59.2)	80 (82.5)	52 (40.9)	<0.001
	Yes	91 (40.8)	17 (17.5)	74 (58.3)	
Primary tumor laterality	Left	123 (55.2)	58 (59.8)	65 (51.2)	0.323
	Right	100 (44.8)	39 (40.2)	61 (48.0)	
Primary tumor lobe	Left upper	83 (37.2)	37 (38.1)	46 (36.5)	0.634
	Left lower	25 (11.2)	13 (13.4)	12 (9.5)	
	Right upper	75 (33.6)	30 (30.9)	45 (35.7)	
	Right middle	18 (8.1)	6 (6.2)	12 (9.5)	
	Right lower	22 (9.9)	11 (11.4)	11 (8.8)	
First-line platinum based therapy	No	5 (2.2)	2 (2.1)	3 (2.4)	0.357
	Carboplatin	72 (32.3)	26 (26.8)	46 (36.2)	
	Cisplatin	146 (65.5)	69 (71.1)	77 (60.6)	
Response to first-line therapy	PD	63 (28.3)	19 (19.6)	44 (34.6)	0.036
	SD	30 (13.4)	11 (11.3)	19 (15.0)	
	PR	115 (51.6)	61 (62.9)	54 (42.5)	
	CR	15 (6.7)	6 (6.2)	9 (7.1)	
Response Assessment	No response ^µ^	63 (28.3)	19 (19.6)	44 (34.6)	0.028
	Disease control ^µ^	160 (71.7)	78 (80.4)	82 (64.6)	
Platinum resistance	Platinum-sensitive	128 (57.4)	65 (67.0)	63 (49.6)	0.027
	Platinum-resistant	95 (42.6)	32 (33.0)	63 (49.6)	
Brain metastasis at progression	No	129 (57.8)	54 (58.7)	75 (57.3)	0.670
	Yes	94 (42.2)	38 (41.3)	56 (42.7)	
NLR	High (≥3.25)	147 (65.9)	39 (41.1)	108 (84.4)	<0.001
	Low (<3.25)	76 (34.1)	56 (58.9)	20 (15.6)	
MLR	High (≥0.32)	140 (62.8)	38 (39.6)	102 (80.3)	<0.001
	Low (<0.32)	83 (37.2)	58 (60.4)	25 (19.7)	
PLR	High (≥148)	125 (56.1)	30 (31.2)	95 (74.8)	<0.001
	Low (<148)	98 (43.9)	66 (68.8)	32 (25.2)	
SII	High (≥885.49)	141 (63.2)	35 (36.5)	106 (83.5)	<0.001
	Low (<885.49)	82 (36.8)	61 (63.5)	21 (16.5)	
SIRI	High (≥1.78)	136 (60.1)	35 (36.1)	101 (80.2)	<0.001
	Low (<1.78)	87 (39.9)	62 (63.9)	25 (19.8)	
PIV	High (≥404.65)	142 (63.7)	37 (38.5)	105 (82.7)	<0.001
	Low (<404.65)	81 (36.3)	59 (61.5)	22 (17.3)	
CAR	High (≥4.50)	114 (51.1)	21 (21.9)	93 (73.2)	<0.001
	Low (<4.50)	109 (48.9)	75 (78.1)	34 (26.8)	
MAR	High (≥118.18)	164 (73.5)	49 (51.1)	115 (90.6)	<0.001
	Low (<118.18)	59 (26.5)	47 (48.9)	12 (9.4)	

Abbreviations: LAR, lactate dehydrogenase-to-albumin ratio; ECOG PS, Eastern Cooperative Oncology Group performance status; PD, progressive disease; SD, stable disease; PR, partial response; CR, complete response; NLR, neutrophil-to-lymphocyte ratio; MLR, monocyte-to-lymphocyte ratio; PLR, platelet-to-lymphocyte ratio; SII, systemic immune-inflammation index; SIRI, systemic inflammatory response index; PIV, pan-immune-inflammation value; CAR, C-reactive protein-to-albumin ratio; MAR, monocyte-to-albumin ratio. ^µ^ No response indicates PD; Disease control rate (DCR) indicates SD, PR, or CR. Footnote: Categorical variables are presented as numbers (%). *p* values refer to comparisons between the low- and high-LAR groups and are shown once per variable. All inflammatory indices were dichotomized using ROC–Youden index-derived cut-off values for 12-month overall survival. Comparisons were performed using the χ^2^ test or Fisher’s exact test, as appropriate.

**Table 2:** Time-dependent ROC analysis of inflammatory indices in extensive-stage small cell lung cancer.

**Index**	AUC(t) 12-mo OS	Youden Cut-Off (OS-12)	Sensitivity	Specificity	AUC(t) 6-mo PFS	DeLong *p* (vs. LAR)
LAR	0.692	65.37	0.825	0.535	0.646	Reference
MAR	0.553	118.18	0.889	0.335	0.573	0.042
NLR	0.547	3.25	0.762	0.497	0.534	0.012
SIRI	0.544	1.78	0.778	0.465	0.527	0.014
PIV	0.541	404.65	0.810	0.445	0.536	0.013
SII	0.535	885.49	0.794	0.503	0.536	0.008
MLR	0.527	0.32	0.762	0.432	0.530	0.008
CAR	0.514	4.50	0.619	0.548	0.534	0.001
PLR	0.498	148.00	0.667	0.465	0.516	0.001

Abbreviations: LAR, lactate dehydrogenase-to-albumin ratio; NLR, neutrophil-to-lymphocyte ratio; PLR, platelet-to-lymphocyte ratio; MLR, monocyte-to-lymphocyte ratio; SII, systemic immune-inflammation index; SIRI, systemic inflammatory response index; PIV, pan-immune-inflammation value; MAR, monocyte-to-albumin ratio; CAR, C-reactive protein-to-albumin ratio. Footnote: Primary horizons: 12-month OS and 6-month PFS. Youden cut-offs were derived from the 12-month OS horizon ROC and applied consistently across endpoints. DeLong p-values compare AUCs for 12-month OS (horizon ROC) using LAR as the reference.

**Table 3:** Univariate Cox regression analyses for OS and PFS in extensive-stage small cell lung cancer.

Variable		Overall Survival		Progression-Free Survival	
		HR (95% CI)	*p*	HR (95% CI)	*p*
Age	per 1-unit increase	1.007 (0.990–1.024)	0.410	1.004 (0.989–1.019)	0.587
Sex	Female vs. Male	1.119 (0.715–1.754)	0.622	1.003 (0.659–1.526)	0.991
ECOG subgroup	≥2 vs. 0–1	1.090 (0.795–1.493)	0.593	1.045 (0.782–1.397)	0.767
Smoking	Yes vs. No	1.062 (0.669–1.684)	0.799	1.217 (0.815–1.818)	0.337
Comorbidity	Yes vs. No	0.819 (0.604–1.110)	0.198	0.917 (0.692–1.217)	0.549
Brain metastasis at diagnosis	Yes vs. No	0.845 (0.619–1.153)	0.288	0.764 (0.573–1.019)	0.067
Liver metastasis at diagnosis	Yes vs. No	0.933 (0.686–1.268)	0.657	0.920 (0.694–1.219)	0.561
Tumor laterality	Right vs. Left	1.189 (0.878–1.610)	0.263	1.019 (0.771–1.346)	0.896
Tumor lobe	Right middle vs. Left upper	2.988 (1.726–5.173)	<0.001	1.852 (1.089–3.150)	0.023
Receipt of platinum (first-line)	Yes vs. No	0.945 (0.372–2.402)	0.906	1.366 (0.543–3.435)	0.507
Response (CR/PR/SD vs. PD)	Disease control vs. PD	0.319 (0.230–0.443)	<0.001	0.267 (0.195–0.366)	<0.001
Platinum resistance	Yes vs. No	3.991 (2.902–5.490)	<0.001	5.861 (4.248–8.088)	<0.001
Brain metastasis at progression	Yes vs. No	0.775 (0.562–1.067)	0.118	0.909 (0.681–1.212)	0.515
NLR	High vs. Low	1.274 (0.938–1.732)	0.122	1.257 (0.949–1.665)	0.110
MLR	High vs. Low	1.252 (0.914–1.715)	0.161	1.199 (0.901–1.595)	0.214
PLR	High vs. Low	1.162 (0.856–1.578)	0.335	1.071 (0.810–1.415)	0.631
SII	High vs. Low	1.336 (0.982–1.818)	0.065	1.189 (0.899–1.574)	0.225
SIRI	High vs. Low	1.322 (0.968–1.806)	0.079	1.241 (0.934–1.647)	0.136
PIV	High vs. Low	1.338 (0.975–1.836)	0.071	1.279 (0.959–1.706)	0.094
MAR	High vs. Low	1.484 (1.046–2.105)	0.027	1.387 (1.012–1.900)	0.042
CAR	High vs. Low	0.971 (0.717–1.314)	0.849	1.045 (0.792–1.379)	0.755
LAR	High vs. Low	1.563 (1.148–2.128)	0.005	1.467 (1.108–1.943)	0.008

Abbreviations: OS, overall survival; PFS, progression-free survival; HR, hazard ratio; CI, confidence interval; ECOG, Eastern Cooperative Oncology Group performance status; PD, progressive disease; SD, stable disease; PR, partial response; CR, complete response; NLR, neutrophil-to-lymphocyte ratio; PLR, platelet-to-lymphocyte ratio; MLR, monocyte-to-lymphocyte ratio; SII, systemic immune-inflammation index; SIRI, systemic inflammatory response index; PIV, pan-immune-inflammation value; MAR, monocyte-to-albumin ratio; CAR, C-reactive protein-to-albumin ratio; LAR, lactate dehydrogenase-to-albumin ratio.

**Table 4:** Multivariable Cox regression analyses for OS and PFS in extensive-stage small cell lung cancer.

Variable	Overall Survival				Progression-Free Survival			
	Model C		Model D		Model C		Model D	
	HR (95% CI)	*p*	HR (95% CI)	*p*	HR (95% CI)	*p*	HR (95% CI)	*p*
Age (per 1-year increase)	1.006 (0.990–1.023)	0.480	1.006 (0.989–1.023)	0.488	1.005 (0.990–1.020)	0.503	1.005 (0.990–1.020)	0.507
ECOG ≥ 2 vs. 0–1	1.070 (0.778–1.472)	0.675	1.072 (0.779–1.474)	0.671	1.019 (0.759–1.367)	0.901	1.019 (0.760–1.367)	0.900
Brain metastasis at diagnosis (Yes vs. No)	0.812 (0.590–1.117)	0.200	0.812 (0.590–1.118)	0.202	0.704 (0.522–0.949)	0.021	0.706 (0.523–0.952)	0.023
Treatment response (Disease control vs. No response)	0.322 (0.230–0.451)	<0.001	0.324 (0.231–0.454)	<0.001	0.262 (0.188–0.366)	<0.001	0.263 (0.189–0.367)	<0.001
LAR (High vs. Low; OS 12-month Youden cut-off = 65.37)	1.425 (1.040–1.954)	0.028	1.406 (0.992–1.994)	0.055	1.165 (0.868–1.564)	0.308	1.146 (0.826–1.591)	0.414
MAR (High vs. Low; OS 12-month Youden cut-off = 118.18)	–	–	1.036 (0.699–1.534)	0.861	–	–	1.041 (0.727–1.491)	0.825

Abbreviations: OS, overall survival; PFS, progression-free survival; HR, hazard ratio; CI, confidence interval; ECOG, Eastern Cooperative Oncology Group performance status; MAR, monocyte-to-albumin ratio; LAR, lactate dehydrogenase-to-albumin ratio.

**Table 5:** Association of pretreatment LDH-to-albumin ratio with adverse treatment-related features in extensive-stage small cell lung cancer.

Outcome	Analysis	Metric	Estimate (95% CI)	*p*
Platinum resistance	ROC	AUC	0.615 (0.541–0.688)	—
		Youden cut-off (LAR)	54.85	—
		Sensitivity	85.3%	—
		Specificity	39.1%	—
	Logistic regression (univariable)	OR (per 1 SD ↑ LAR)	1.95 (1.27–2.99)	0.002
	Logistic regression (multivariable *)	aOR (per 1 SD ↑ LAR)	2.31 (1.41–3.81)	0.001
Lack of objective treatment benefit from first-line therapy	ROC	AUC	0.648 (0.566–0.726)	—
		Youden cut-off (LAR)	57.75	—
		Sensitivity	85.7%	—
		Specificity	41.9%	—
	Logistic regression (univariable)	OR (per 1 SD ↑ LAR)	1.96 (1.30–2.96)	0.001
	Logistic regression (multivariable *)	aOR (per 1 SD ↑ LAR)	2.04 (1.33–3.14)	0.001

Abbreviations: LAR, lactate dehydrogenase-to-albumin ratio; AUC, area under the curve; OR, odds ratio; aOR, adjusted odds ratio. Lack of objective treatment benefit was defined as progressive disease as the best response to first-line therapy. * Multivariate models were adjusted for age, ECOG performance status, and presence of brain metastasis at diagnosis. ↑ indicates increase per 1 standard deviation.

## Data Availability

The datasets generated and analyzed during the current study are available from the corresponding author upon reasonable request, subject to approval by the Medical Oncology Department of the University of Health Sciences Antalya Training and Research Hospital.

## Supplementary Materials

The following supporting information can be downloaded at https://www.mdpi.com/article/10.3390/jcm15093353/s1, Table S1: Definitions and Formulas of Biomarker Indices.

## Abbreviations

The following abbreviations are used in this manuscript:

Abbreviation	Definition
aOR	Adjusted odds ratio
AUC	Area under the curve
CAR	C-reactive protein-to-albumin ratio
CI	Confidence interval
CR	Complete response
ECOG PS	Eastern Cooperative Oncology Group performance status
HR	Hazard ratio
LAR	Lactate dehydrogenase-to-albumin ratio
MAR	Monocyte-to-albumin ratio
MLR	Monocyte-to-lymphocyte ratio
NLR	Neutrophil-to-lymphocyte ratio
OR	Odds ratio
OS	Overall survival
PD	Progressive disease
PFS	Progression-free survival
PIV	Pan-immune inflammation value
PLR	Platelet-to-lymphocyte ratio
PR	Partial response
SCLC	Small cell lung cancer
SD	Stable disease
SII	Systemic immune-inflammation index
SIRI	Systemic inflammatory response index
