# Supplementary material for: Pretreatment Lactate Dehydrogenase-to-Albumin Ratio and Clinical Outcomes in Extensive-Stage Small Cell Lung Cancer: A Multicenter Real-World Study
Source: J Clin Med. 2026 Apr 28;15(9):3353. doi: 10.3390/jcm15093353 (PMC13163826; doi:10.3390/jcm15093353)
Supplement: Supplementary file 1 [file jcm-15-03353-s001.zip › jcm-4241863-supplementary.pdf]

Supplementary Table S1. Definitions and Formulas of Biomarker Indices.

Index	Full Name	Formula	Components
LAR	Lactate Dehydrogenase-to-Albumin Ratio	LDH (U/L) / Albumin (g/dL)	Serum LDH, Albumin
NLR	Neutrophil-to-Lymphocyte Ratio	Neutrophil count / Lymphocyte count	Absolute neutrophil, lymphocyte counts
MLR	Monocyte-to-Lymphocyte Ratio	Monocyte count / Lymphocyte count	Absolute monocyte, lymphocyte counts
PLR	Platelet-to-Lymphocyte Ratio	Platelet count / Lymphocyte count	Platelet, lymphocyte counts
SII	Systemic Immune-Inflammation Index	(Platelet × Neutrophil) / Lymphocyte	Platelet, neutrophil, lymphocyte counts
SIRI	Systemic Inflammatory Response Index	(Monocyte × Neutrophil) / Lymphocyte	Monocyte, neutrophil, lymphocyte counts
PIV	Pan-Immune-Inflammation Value	(Platelet × Neutrophil × Monocyte) / Lymphocyte	Platelet, neutrophil, monocyte, lymphocyte counts
CAR	C-Reactive Protein-to-Albumin Ratio	CRP (mg/L) / Albumin (g/dL)	Serum CRP, Albumin
MAR	Monocyte-to-Albumin Ratio	Monocyte count / Albumin (g/dL)	Monocyte count, Albumin
